# Truncated O‐glycans promote epithelial‐to‐mesenchymal transition and stemness properties of pancreatic cancer cells

**DOI:** 10.1111/jcmm.14572

**Published:** 2019-08-07

**Authors:** Divya Thomas, Satish Sagar, Thomas Caffrey, Paul M. Grandgenett, Prakash Radhakrishnan

**Affiliations:** ^1^ Eppley Institute for Research in Cancer and Allied Diseases, Fred & Pamela Buffett Cancer Center University of Nebraska Medical Center Omaha NE USA

**Keywords:** core‐1 synthase, COSMC, EMT, PDAC, stem cells, truncated O‐glycans

## Abstract

Aberrant expression of Sialyl‐Tn (STn) antigen correlates with poor prognosis and reduced patient survival. We demonstrated that expression of Tn and STn in pancreatic ductal adenocarcinoma (PDAC) is due to hypermethylation of Core 1 synthase specific molecular chaperone (COSMC) and enhanced the malignant properties of PDAC cells with an unknown mechanism. To explore the mechanism, we have genetically deleted COSMC in PDAC cells to express truncated O‐glycans (SimpleCells, SC) which enhanced cell migration and invasion. Since epithelial‐to‐mesenchymal transition (EMT) play a vital role in metastasis, we have analysed the induction of EMT in SC cells. Expressions of the mesenchymal markers were significantly high in SC cells as compared to WT cells. Equally, we found reduced expressions of the epithelial markers in SC cells. Re‐expression of COSMC in SC cells reversed the induction of EMT. In addition to this, we also observed an increased cancer stem cell population in SC cells. Furthermore, orthotopic implantation of T3M4 SC cells into athymic nude mice resulted in significantly larger tumours and reduced animal survival. Altogether, these results suggest that aberrant expression of truncated O‐glycans in PDAC cells enhances the tumour aggressiveness through the induction of EMT and stemness properties.

## 1 INTRODUCTION

Pancreatic ductal adenocarcinoma (PDAC) is one of the most devastating and lethal malignancies worldwide with a predicted incidence of ~420 000 and about equally associated mortality of ~410 000 by 2020.^1^ Identification of novel biomarkers for the early disease detection remains as one of the major challenges to overcome the existing difficulties in the treatment modalities for PDAC. Sialyl‐Tn antigen (Neu5Acα2‐6 GalNAcα‐O‐Ser/Thr, STn), the simple mucin‐type carbohydrate antigen, has attracted much research attention due to its abundant expression in the PDAC, ovarian, colorectal and most of the gastrointestinal carcinomas.^2^, ^3^, ^4^, ^5^ Furthermore, expression of STn antigen correlates with poor prognosis, whereas its expression is never found in the normal pancreas,^6^ which highlights STn antigen as a possible target for therapeutic intervention against PDAC progression. There are several factors that contribute to the formation of STn antigen on glycoproteins. Previously, we found that hypermethylation of COSMC is one of the prevalent causes for the expression of the immature truncated O‐glycans (Tn and STn antigens) in ~40% of the PDAC tumours.^7^ Further, COSMC is the molecular chaperone for Core 1 synthase (C1GalT1) that stabilizes the enzyme.^8^ Loss of COSMC and aberrant expression of truncated O‐glycans induce oncogenic features, including compromised adhesion, increased cell migration, reduced apoptosis, loss of tissue architecture, invasive growth and metastasis.^7^, ^9^ However, the molecular mechanism whereby these truncated O‐glycans enhance the tumorigenic properties of PDAC cells remains elusive.

Studies have demonstrated that epithelial‐to‐mesenchymal transition (EMT) is a critical phenomenon for tumour metastasis where switching of epithelial phenotype to mesenchymal phenotype facilitates the tumour cell migration through the matrix to the distant organ sites. This orchestrated biological event is complemented by the down‐regulation of the epithelial markers, such as E‐cadherin, claudin and occludin, and up‐regulation of mesenchymal markers, such as N‐cadherin, vimentin, transcription factors Snail and Slug.^10^, ^11^ In human PDAC, EMT is closely associated with the invasive tumour, development of metastasis and negatively affect overall patient survival.^12^ A recent study has demonstrated that overexpression of N‐acetylgalactosaminyl transferase 6 (GALNT6) a glycosyltransferase involved in O‐glycosylation potentially disrupts cell morphogenesis and induced cellular changes similar to EMT in mammary epithelial cells.^13^ Further, hyperglycemia, especially O‐glycosylation induced EMT in lung adenocarcinoma cells to promote tumour metastasis has also been reported.^14^

In the light of these reports, we hypothesized the possible role of EMT as the underlying mechanism for the enhanced tumorigenic feature of truncated O‐glycans expressing COSMC deleted PDAC cells. Here, we show for the first time that COSMC deletion induced aberrant expression of immature truncated O‐glycans enhances the PDAC tumorigenesis through promoting EMT and stemness properties of PDAC cells.

## 2 MATERIALS AND METHODS

### 2.1 Human Rapid Autopsy PDAC samples

De‐identified matched sets of human primary pancreatic tumour (n = 6) and metastatic lesions (n = 6), and unaffected normal pancreas tissues (n = 6) were procured from the Rapid Autopsy Pancreas Program tissue bank at the University of Nebraska Medical Center (UNMC).

### 2.2 Human PDAC Cells and transfection

Human PDAC cells T3M4 (wild‐type, WT), T3M4 COSMC Knockout (SimpleCells, SC), T3M4 COSMC re‐expression (SC‐R), Capan‐2 (WT) and Capan‐2 (SC) cells were generated, characterized and cultured as described previously.^7^, ^15^ Briefly, COSMC re‐expression in PDAC cells was performed by amplification of full‐length COSMC cDNA by using the primers F‐5′‐CGTGAGAGGAAACCCGTG‐3′ and R‐5′‐TGTGTGGTTATACCAGTGCC‐3′. The verified cDNA was cloned into the pLVX‐Puro lentiviral vector expression system (Clone tech) and then packaged in HT293A packaging cells. The COSMC and vector control lentiviral particles were then transduced into T3M4 SC cells. Clones were selected using puromycin (3 μg/mL; Invivogen).

### 2.3 PCR analysis of COSMC/C1GALT1C1 and Core 1 synthase/C1GALT1

Total RNA was isolated from T3M4 and Capan‐2 (WT, SC and SC‐R) cells, and cDNA was synthesized using Verso cDNA synthesis kit (Thermo Fisher Scientific) according to the manufacturer's instruction. C1GALT1C1 (COSMC), C1GALT1 (Core‐1 synthase) and GAPDH genes were amplified using cDNA‐specific primers (Table S1). PCR was carried out at 95°C for 3 minutes followed by 30 cycles of 95°C for 30 seconds, 55°C for 30 seconds and 72°C for 1 minutes followed by a single incubation at 72°C for 5 minutes. PCR products were resolved by electrophoresis on a 1.2% agarose gel.

### 2.4 In vitro invasion and migration assays

Invasion and migration analyses were performed as described previously.^7^ Briefly, T3M4 WT and SC cells (5 × 10^4^) were seeded on top portion of the polyethylene terephthalate inserts and matrigel‐coated Boyden chambers (BD Biosciences). The cells that migrated or invaded through the membrane barrier were fixed, stained and counted in five different fields using a light microscope (20×). The number of invaded or migrated cells was expressed as the mean number of cells that transited to the lower part of the membrane. These experiments were performed in triplicates.

### 2.5 Immunofluorescence analysis

Standard immunofluorescence staining procedure was performed for STn antigen expression by anti‐STn mAb TKH2 and Vicia Villosa Agglutinin (VVA) in PDAC (T3M4 and Capan‐2) WT, SC and SC‐R cells. Briefly, cells were fixed with 4% paraformaldehyde, permeabilized with 0.15% Triton X‐100 in 1× PBS containing 1% BSA and were incubated with mouse anti‐TKH2 (a kind gift from Dr Ulla Mandel, University of Copenhagen, Denmark) and fluorescein labelled VVA (FL‐1231, Vector Laboratories) for 2 hours at room temperature. Cells incubated with mAb TKH2 were washed and stained with Alexa Fluor 488‐conjugated goat antimouse IgG (Jackson ImmunoResearch). Finally, cells were washed and mounted with Vectashield mounting medium containing DAPI (Vector Laboratories) and images were captured using Zeiss LSM 710 confocal laser scanning microscope (Carl Zeiss, Inc).

### 2.6 Antibody labelling and Fluorescence‐activated cell sorting analysis

For side cell population analysis, T3M4 WT and SC cells (1 × 10^6^ cells/mL) were re‐suspended in DMEM supplemented with 2% foetal bovine serum (FBS) at 37°C. Then, the cells were labelled with Hoechst 33342 (Thermo Fisher Scientific) at a concentration of 5 µg/mL for 2h at 37°C in dark with gentle agitation with or without verapamil (50 µg/mL). The cells were centrifuged at 500 *g* for 5 minutes at 4°C; re‐suspended in ice‐cold PBS containing 2% FBS. Cells were counterstained with 5 µg/mL propidium iodide (Sigma‐Aldrich), and cell sorting was performed using a FACS Vantage flow cytometer (BD Biosciences, LSRII). Propidium iodide‐positive dead cells and debris were excluded. For the analysis of CD133‐positive cell population, T3M4 and Capan‐2 (WT and SC) cells (1 × 10^6^ cells/mL) were incubated with phycoerythrin‐conjugated CD133 (PE‐CD133) for 30 minutes at 4°C in dark and analysed in a flow cytometer. These experiments were performed in triplicate. For the analysis of VVA and CD44, T3M4 and Capan‐2 (WT, SC and SC‐R) cells (1 × 10^6^) were stained with fluorescein labelled VVA (FL‐1231, Vector Laboratories) and rabbit anti‐CD44 (Abcam), respectively, for 30 minutes. CD44 incubated cells were further stained with Alexa Fluor 488‐conjugated goat anti‐rabbit IgG (Jackson ImmunoResearch). Flow cytometry analysis was performed on FACS (BD Biosciences, LSRII). The Flowjo software was used to analyze the data.

### 2.7 Orthotopic pancreas tumour model and animal survival

T3M4 WT and SC cells were orthotopically implanted into the mice pancreas as described earlier.^16^ Briefly, cells (0.25 × 10^6^/30 µL PBS) were orthotopically implanted into the pancreas of athymic nu/nu mice (Crl:NU‐Foxn1^nu^) (n = 13/group). After 28 days of implantation, the animals were killed and the tumour weight, volume and incidence of metastases were determined. For animal survival analyses, the same experiment was performed in athymic nude mice (n = 15/group). Animal survival was monitored on a daily basis, or animals were killed at a pre‐determined end‐point, if the tumour has been grown more than 2 cm in diameter. All the animals were housed under standard housing conditions at the University of Nebraska Medical Center animal core facilities. Animal procedures included in this study were reviewed and approved by the UNMC institutional animal care and use committees (IACUC).

### 2.8 Western blot analysis

Cell lysates were prepared from T3M4 (WT, SC and SC‐R) and Capan 2 (WT and SC) cells. For mouse tissue sample, the tissue homogenate was prepared in homogenizing buffer. 30 μg of proteins were resolved in a gradient (4%‐20%) denaturing polyacrylamide gel (Bio‐Rad) and transferred to polyvinylidene difluoride (PVDF) membranes (Millipore). After blocking with 5% BSA, the membranes were incubated with the respective primary antibodies (Table S2). After incubation with HRP‐conjugated secondary antibodies, the antigen‐antibody complex was developed using Bio‐Rad enhanced chemiluminescence (ECL) Prime Western Blotting detection reagent (General Electric Healthcare Life Sciences).

### 2.9 Immunohistochemistry

For the analysis of STn antigen expression in RAP samples, the paraffin‐embedded tissue sections were deparaffinized with xylene, hydrated with series of ethanol and quenched with hydrogen peroxide. Antigen retrieval was performed with citrate buffer (pH 6.0); blocked with universal blocker (Thermo fisher Scientific) and incubated with TKH2 monoclonal antibody (a kind gift from Dr Ulla Mandel, University of Copenhagen, Denmark) for 2 hours at room temperature. For the analysis of E‐cadherin, N‐cadherin and CD 133 in mouse tissue sections, the paraffin‐embedded slides were processed as describes above and incubated with rabbit anti‐N‐cadherin (ab18203), rabbit anti‐E‐cadherin (ab15148) and Rabbit anti‐CD 133 (ab16518). The slides were washed and incubated with HRP‐conjugated secondary antibody. After 1 hour, the slides were washed with TBST, treated with 3,3′‐diaminobenzidine tetrahydrochloride (DAB) substrate (SK‐4105) and counter stained with haematoxylin. For the lectin‐based detection of glycoproteins, the deparaffinized and re‐hydrated tissue sections were blocked with Carbp‐Free™ blocking solution (SP‐5040) for 30 minutes and incubated with biotinylated lectin (10 μg/mL in PBS) for 30 minutes at room temperature. The sections were incubated further with VECTASTAIN ABC complex (peroxidase, PK‐6100) for 30 minutes at room temperature and developed using DAB according to the kit instructions. All the slides were dehydrated with ethanol series, and after xylene washes, the slides were mounted with coverslip. The protein expression was analysed by a pathologist. The histological scoring was performed based on stain proportion (0%‐100%) and intensity (0‐negligible, 1‐low, 2‐moderate and 3‐high). The histoscore was generated by multiplying the stain proportion score (1 = <5%, 2 = 5%‐25%, 3 = 26%‐50%, 4 = 51%‐75%, 5 = >75%) with the intensity score (0‐3) to obtain values between 0 and 15.

### 2.10 Tumour sphere formation assay

T3M4 (WT, SC and SC‐R) and Capan‐2 (WT, SC) cells (1 × 10^4^) were cultured in a Corning^®^ Costar^®^ Ultra‐low attachment multiwell plate (CLS3471) containing 3dGRO™ medium (S3077, Sigma) for 4‐10 days. The sphere passage was administrated every 5‐8 days. The tumour sphere numbers were counted under phase‐contrast microscope at 10× magnification.

### 2.11 Statistical analysis

In vitro cell migration and invasion was analysed between the groups by unpaired *t* test. Protein expressions by Western blotting were quantified using ImageJ software, and the statistical significance between groups was analysed by two‐way ANOVA. The significance of tumour weight, volume and metastasis between the groups was calculated by two‐tailed Student's *t* test and two‐tailed Fisher's exact test. Animal survival rates were graphed using Kaplan‐Meier method, and the statistical significance between the groups was calculated by log‐rank statistical analysis. *P* value < .05 was considered as statistically significant.

## 3 RESULTS

### 3.1 Expression of Sialyl‐Tn antigen in primary and metastatic PDAC tumour tissues

**Figure 1:**
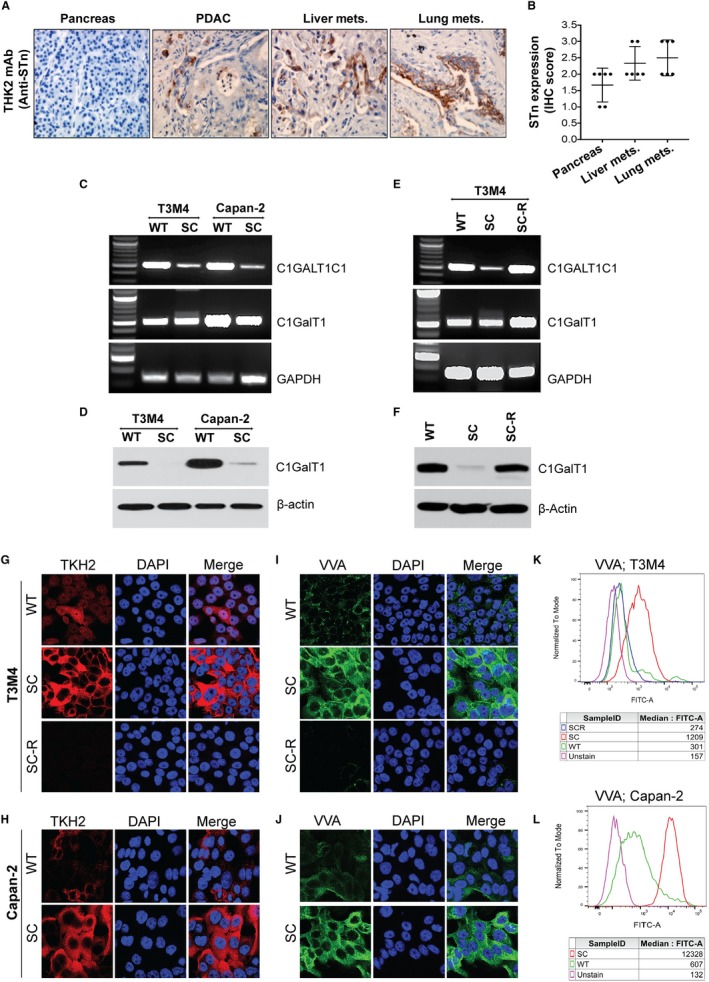
STn antigen enhances PDAC tumorigenicity: A, IHC analysis of STn antigen expression in human RAP PDAC tumour tissues (normal pancreas, primary, liver mets and lung mets) using TKH2 monoclonal antibody. The images were taken at a magnification of 20×. B, IHC intensity score was compared against each tumour samples (n = 6). C, PCR amplification of COSMC/C1GALT1C1 and Core 1 synthase/C1GALT1 in T3M4 and Capan‐2 (WT and SC) cells. D, Western blotting of Core 1 synthase (C1GalT1) in T3M4 and Capan‐2 (WT and SC) cells. E, PCR amplification of COSMC/C1GALT1C1 and Core 1 synthase/C1GALT1 gene in T3M4 WT, SC and COSMC re‐expression (SC‐R) cells. Amplification of GAPDH was used as an internal loading control. F, Western blotting of Core 1 synthase (C1GalT1) in T3M4 WT, SC and SC‐R cells. Immunofluorescence analysis of STn antigen expression by TKH2 antibody in T3M4 WT, SC and SC‐R cells (G) and Capan‐2 WT and SC cells (H). Alexa fluor 488‐conjugated secondary antibody was used (images are represented with pseudo red‐colour). Magnification: 63×; Scale bar: 10 µm. Immunofluorescence analysis of VVA in T3M4 WT, SC and SC‐R cells (I) and Capan‐2 WT and SC cells (J). Alexa fluor 488‐conjugated secondary antibody was used. Magnification: 63×; Scale bar: 10 µm. Analysis of VVA by flow cytometry in T3M4 WT, SC and SC‐R cells (K) and Capan‐2 WT and SC cells (L)

We have assessed the clinical relevance of Sialyl‐Tn (STn) antigen in PDAC by analysing its expression on tissues of normal pancreas (n = 6), primary pancreatic tumour (n = 6), liver (n = 6) and lung (n = 6) metastases (obtained from PDAC patients who underwent rapid autopsies) by immunohistochemistry (IHC). Consistent with available reports, we found no expression of STn antigen in normal pancreas. However, aberrant expression of STn antigen was detected in primary tumour. Interestingly, STn antigen is more highly expressed in liver as well as lung metastases (Figure 1A). Figure 1B shows the graphic representation of IHC intensity score for the relative expression of STn antigen in each autopsy patient's primary tumour, liver mets and lung mets samples.

### 3.2 Genetic deletion of COSMC enhances tumorigenic features in PDAC cells

**Figure 2:**
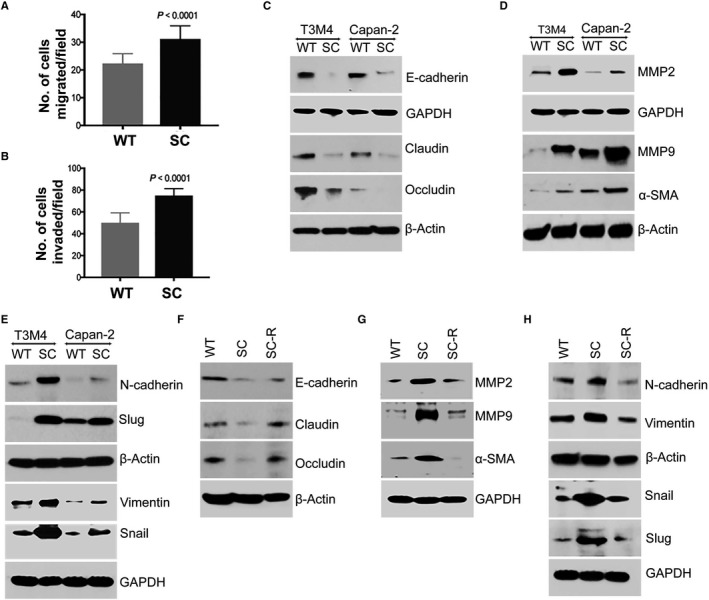
Truncated O‐glycans enhances PDAC cell invasiveness and induction of EMT. A, Migration assay. T3M4 WT and SC cells (5 × 10^4^) that migrate through trans well inserts were fixed and counted five different fields under the light microscope (20×) (n = 3). B, Matrigel Invasion assay. T3M4 WT and SC cells (5 × 10^4^) that invaded through matrigel‐coated Boyden chambers were fixed and counted five different fields under the microscope (20×) (n = 3). *P* value < 0.05 considered as statistically significant. C, Western blotting of T3M4 and Capan‐2 (WT and SC) cell lysates with epithelial marker proteins E‐cadherin, claudin and occludin. D, Western blotting of T3M4 and Capan‐2 (WT and SC) cell lysate with MMP2, MMP9 and α‐SMA. E, Western blotting of T3M4 and Capan‐2 (WT and SC) cell lysate with mesenchymal marker proteins N‐cadherin, vimentin and transcription factors Snail and Slug. F, Western blotting of epithelial marker proteins E‐cadherin, claudin and occludin in T3M4 WT, SC and SC‐R. G, Western blotting of MMP2, MMP9 and α‐SMA in T3M4 WT, SC and SC‐R cells. H, Western blotting of mesenchymal marker proteins N‐cadherin, vimentin and transcription factors Snail and slug in T3M4 WT, SC and SC‐R cells. Detection of β‐actin and GAPDH served as loading control

We previously demonstrated that reduced expression of C1GalT1 and/or hypermethylation of its molecular chaperone COSMC are the major causative factors for the truncation of mucin‐type O‐glycosylation in clinical specimens of PDAC.^7^ In continuation with the previous work, to explore the biological role of truncated O‐glycans, we have utilized COSMC knockout PDAC cells (T3M4 and Capan‐2). Disruption of COSMC and loss of its expression in T3M4 and Capan‐2 were observed by PCR (Figure 1C), while the mRNA expression of C1GALT1 (core‐1 synthase) remained same in both T3M4 and Capan‐2 WT and SC cells (Figure 1C) which is in accordance with the previous reports.^7^, ^9^ We further examined the COSMC deletion induced degradation of C1GalT1 protein in PDAC SC cells (Figure 1D). To confirm these results, we re‐expressed the COSMC in T3M4 SC cells and confirmed at the mRNA levels by PCR (Figure 1E) and protein levels by Western blot (Figure 1F). We further validated these results by analysing the expression of STn antigen in isogenic PDAC cells (WT, SC and SC‐R). T3M4 SC and Capan‐2 SC cells exhibited abundant expression of STn antigen, whereas WT cells showed negligible expression of STn antigen, which was totally absent in T3M4 SC‐R cells (Figure 1G,H). Additionally, immunofluorescence analysis of VVA in T3M4 and Capan‐2 cells revealed that SC cells exhibited more STn antigen expression (Figure 1I,J) which was negligible or absent in WT as well as SC‐R cells (Figure 1I,J). Flowcytometric analysis of VVA further confirmed this observation (Figure 1K,L). Next, we investigated the impact of COSMC deletion and subsequent expression of truncated O‐glycans in the invasive potential of PDAC cells. Figure 2A demonstrates a significant increase in the migration of T3M4 SC cells through the matrigel chamber as compared to T3M4 WT cells (*P* < .001). In accordance with this, SC cells also exhibited a significantly increased invasive capacity than WT cells (*P* < .001) (Figure 2B). These results confirm that COSMC deletion mediated truncation of O‐glycans directly promotes the oncogenic features of PDAC cells.

### 3.3 COSMC deletion promotes PDAC tumorigenicity through the induction of epithelial‐to‐mesenchymal transition

The observed increased invasiveness and migration of T3M4 cells upon COSMC deletion further prompted us to decipher the mechanism by which this truncated O‐glycan enhances the oncogenic features in PDAC cells. Several studies have shown the close association between EMT and cancer metastasis.^17^, ^18^ To examine whether cancer‐specific truncation of O‐glycans has any role in the induction of EMT, we have analysed the expression of epithelial as well as mesenchymal markers in isogenic PDAC cells, T3M4 (WT and SC) and Capan‐2 (WT and SC). Decreased expression of E‐cadherin was observed in T3M4 SC (*P* = 0.0188) and Capan‐2 SC (*P* = 0.0131) as compared to their WT cells (Figure 2C and Figure S1A). Also, reduced expression of claudin was detected in T3M4 SC (*P* = 0.0015) and Capan‐2 SC (*P* = 0.0063) when compared with their isogenic WT cells (Figure 2C and Figure S1A). In addition to this, we also observed a decreased expression of occludin in T3M4 SC cells (*P* = 0.0067) as compared to T3M4 WT cells (Figure 2C and Figure S1A). Interestingly, either low or no expression of occludin was seen in Capan‐2 SC cells as compared to Capan‐2 WT cells (Figure 2C and Figure S1A). Previous studies have reported that matrix degrading enzymes cleave the stromal basement epithelium of extracellular matrix (ECM) and therefore facilitates the invasion of transmitted cells.^11^ We, therefore, analysed the expression of Matrix metalloproteinases (MMPs) 2 and 9 in WT and SC cells. COSMC deletion enhanced the expression of MMP2 and MMP9 in T3M4 SC (*P* = 0.0005 and *P* < 0.0001, respectively) and Capan‐2 SC (*P* = 0.0029 and *P* = 0.0025, respectively) cells as compared to their WT cells (Figure 2D and Figure S1B). Next, we analysed the expression of alpha smooth muscle actin (α‐SMA), a commonly used marker for the cells in the transition phase.^19^ T3M4 SC and Capan‐2 SC cells showed an increased expression of α‐SMA (*P* < 0.0004 and *P* = 0.0007, respectively) as compared to their WT cells (Figure 2D and Figure S1B). In order to further explore whether mesenchymal transition is ensued, we have analysed the expression of major mesenchymal markers such as N‐cadherin, vimentin and the transcription factors Snail and Slug in PDAC cells. We detected a significant fold increase in the expression of mesenchymal markers N‐cadherin and vimentin in T3M4 SC (*P* = .0005 and *P* = 0.0161, respectively) and Capan‐2 SC (*P* = 0.0005 and *P* = 0.0346, respectively) cells as compared to WT cells (Figure 2E and Figure S1C). Also, in the protein expression of the transcription factor Snail showed a significant higher expression in T3M4 SC (*P* = 0.0062) and Capan‐2 SC (*P* = 0.0357) cells, respectively, as compared with their WT cells (Figure 2E and Figure S1C). Additionally, we observed an increased expression of Slug in T3M4 SC (*P* = 0.0004) and Capan‐2 SC (*P* = 0.0007) as compared with their isogenic WT cells (Figure 2E and Figure S1C). Interestingly, the induction of EMT upon COSMC deletion was found to be reversed in COSMC re‐expressed PDAC cells (SC‐R). The expressions of epithelial markers E‐cadherin (*P* = 0.0035), claudin (*P* = 0.0006) and occludin (*P* = 0.0007) were high in T3M4 SC‐R cells when compared to T3M4 SC cells (Figure 2F and Figure S1D). Concomitantly, the protein expressions of MMP2 (*P* = 0.0494), MMP9 (*P* = 0.0052), α‐SMA (low or no expression) and mesenchymal‐specific markers N‐cadherin (*P* = 0.0188), vimentin (*P* = 0.0051) Snail (*P* = 0.0142) and Slug (*P* = 0.0025) were significantly less in SC‐R cells compared to T3M4 SC cells (Figure 2G,H and Figure S1E,F). These results indicate that truncation of O‐glycan exacerbates malignant features in PDAC cells through the induction of EMT.

### 3.4 Truncation of O‐glycans enhance the stemness feature of PDAC cells

**Figure 3:**
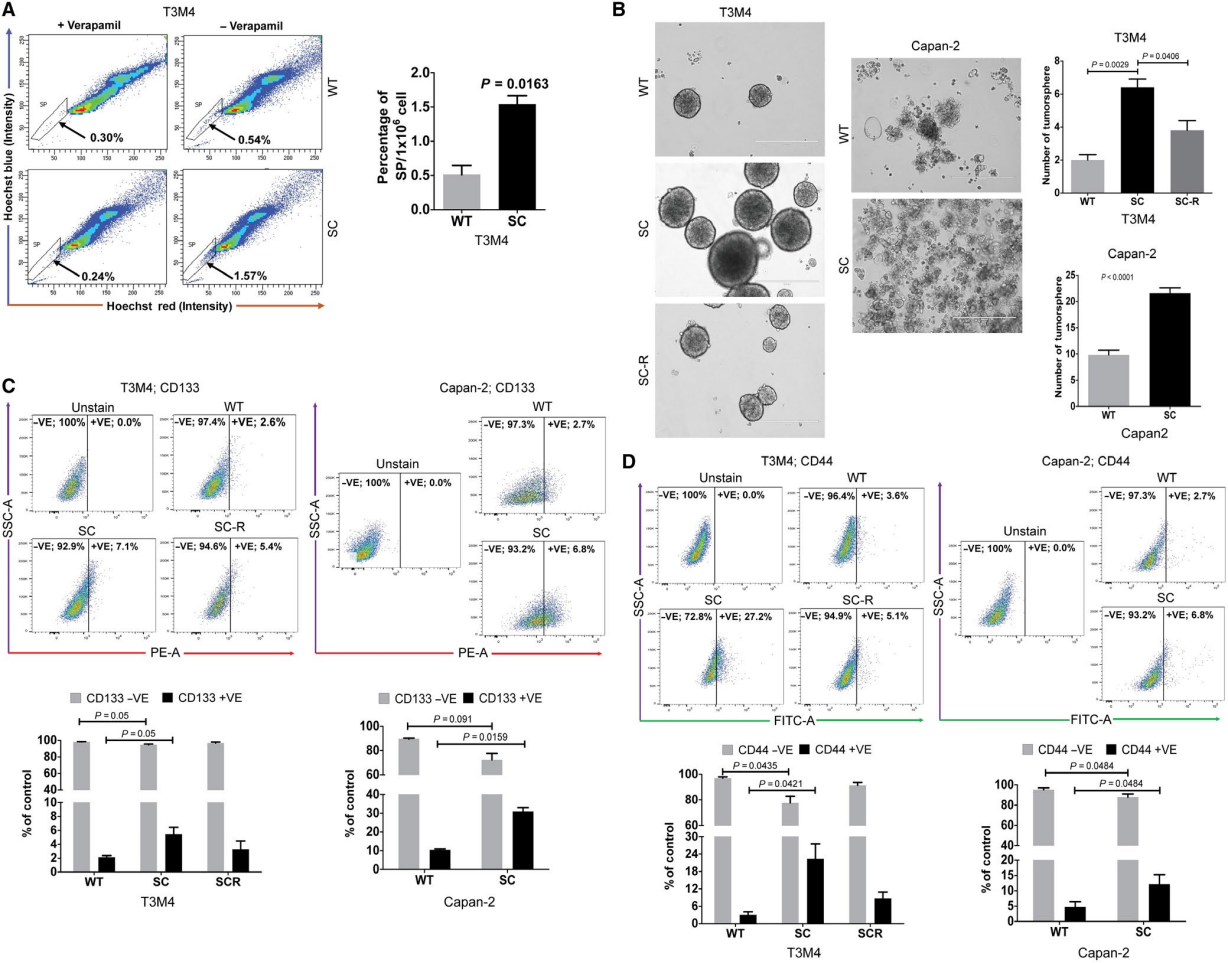
Truncated O‐glycans induce stemness in PDAC cells. A, T3M4 cells (WT and SC, 1 × 10^6^ cells/mL) were stained with Hoechst 33342 with or without verapamil and analysed by flow cytometry. The side population (SP) cells, which appear in the absence of verapamil, are outlined and shown as a percentage of the total cell population (left panel). The graphical representation of respective side cell population in T3M4 WT and SC cells (n = 3) (right panel). B, Tumour sphere forming assay. T3M4 (WT, SC and SC‐R) and Capan‐2 (WT and SC) Cells cultured in ultra‐low attachment plates with 3dGRO™ medium were analysed for the ability to form tumour sphere. COMC knockout enhanced tumour sphere formation in PDAC cells. C, Flow cytometry analysis of CD44 expression. T3M4 (WT, SC and SC‐R) and Capan‐2 (WT and SC) cells (1 × 10^6^ cells/mL) were incubated with rabbit anti‐CD44 antibody and stained with Alexa fluor 488‐conjugated anti‐rabbit IgG and analysed in a flow cytometer. D, Flow cytometry of CD133 expression. T3M4 (WT, SC and SC‐R) and Capan‐2 (WT and SC) cells (1 × 10^6^ cells/mL) were incubated with phycoerythrin‐conjugated CD133 and analysed in flow cytometer. The graphical representation of the expression of CD 44 and CD133 in PDAC cells (n = 3) (bottom panel). *P* value < .05 considered as statistically significant

It is well known that PDAC is composed of both undifferentiated or poorly differentiated cancer stem cells and more differentiated malignant cells that are derived from cancer stem cells.^20^ Since we found that cancer‐specific truncation of O‐glycans enhanced the oncogenic features through the induction of EMT, we sought to explore the possibility of truncated O‐glycans induced stem cell side populations in PDAC cells as EMT is closely associated with cellular plasticity.^21^ Flow cytometry analysis for the side cell population with Hoechst 33342 dye in T3M4 WT and SC cells (1 × 10^6^) is depicted in Figure 3A. Interestingly, a significant increase in the percentage of side cell population (SP) was observed in T3M4 SC cells (1.57% ± 0.22) when compared with WT cells (0.54% ± 0.05) (*P* = 0.0163). SP fraction of cells is known to vanish upon the treatment with ATP‐binding cassette (ABC) transporters inhibitors such as verapamil.^22^ Treatment of cells in the presence and absence of verapamil clearly indicate the more percentage of SP in SC cells. Further, we found that T3M4 SC and Capan‐2 SC cells have the ability to form more tumour spheres than its isogenic WT cells (*P* = .0029 and *P* < .0001, respectively) (Figure 3B). Re‐expression of COSMC in SC cells significantly reduced the ability to form tumour spheres (*P* = 0.0406) (Figure 3B). To further confirm our hypothesis that truncated O‐glycans enhances the stemness of PDAC cells, we have analysed the relative percentages of cells expressing CD44 and CD133.^23^ As shown in Figure 3C, T3M4 SC cells exhibited more percentage of CD44‐positive cells (*P* = 0.0421) as compared to WT cells. Further, COSMC re‐expression in SC cells reduced the percentage of CD44‐positive cells (Figure 3C). The percentage of CD44‐positive cells were more in Capan‐2 SC cells (*P* = 0.0484) as compared to WT cells (Figure 3C). Simultaneously, it was observed that T3M4 SC cells exhibit more CD133‐positive cells (*P* = 0.05) as compared to WT and SC‐R cells (Figure 3D). A similar trend was observed in Capan‐2 cells where SC cells exhibited more percentage of CD133 positive cells (*P* = 0.0159) as compared to WT cells (Figure 3D). Altogether, these results suggest that aberrant expression of STn antigen increases the number of cells with self‐renewal capacity in SC populations.

### 3.5 Truncated O‐glycans modulate PDAC cells in vivo tumour growth and animal survival

**Figure 4:**
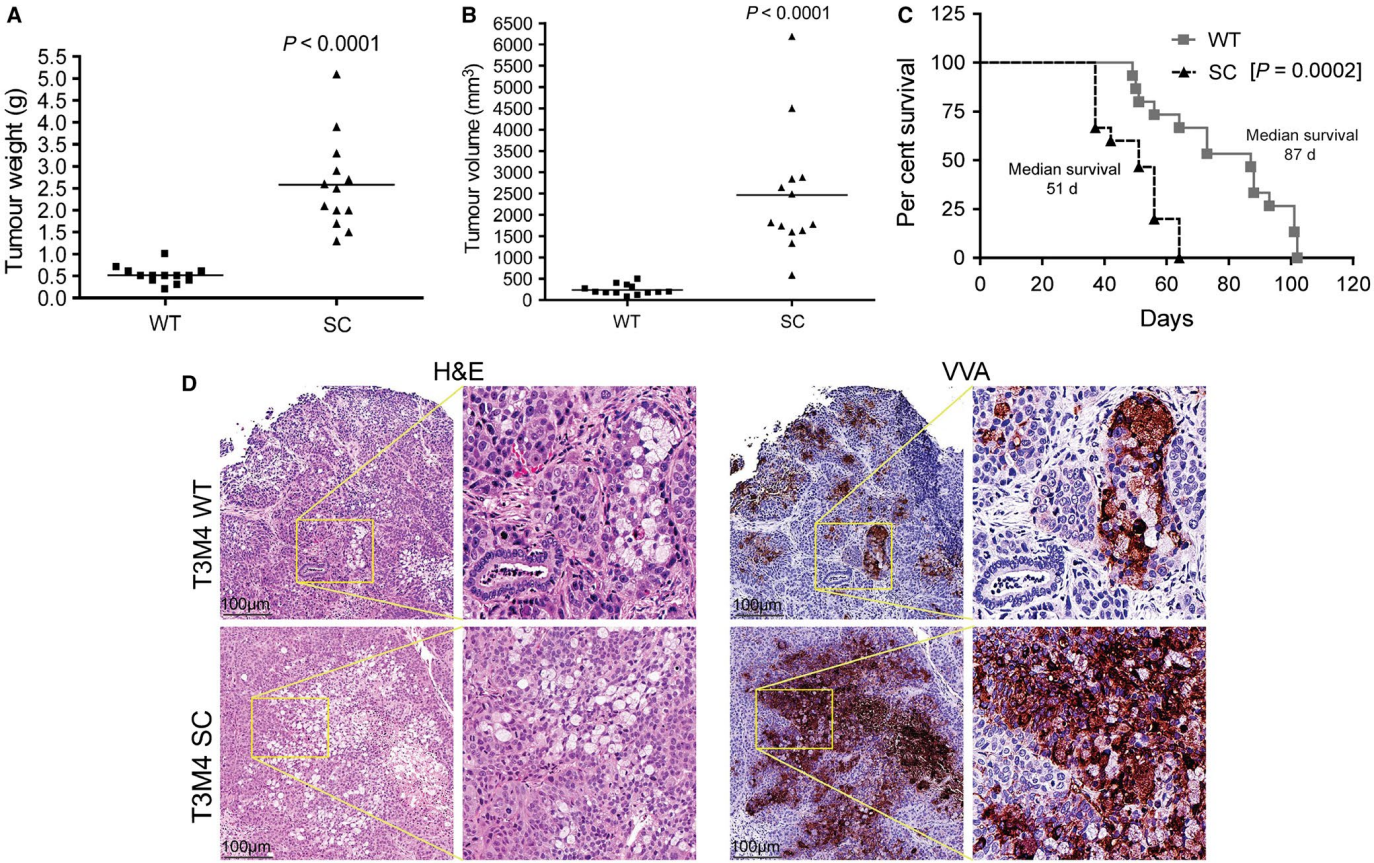
Truncated O‐glycans induced tumour malignancy. Orthotopic implantation of T3M4 WT and COSMC deleted cells (0.25 × 10^6^/30 µL PBS) in pancreas of athymic nude mice (n = 13). After 28 days of post‐injection, the tumour weight (g) (A) and tumour volume (B) was measured. C, Survival of tumour‐bearing animals. Orthotopic implantation of T3M4 WT and SC cells into the pancreas of athymic nude mice (n = 15/group). The Kaplan‐Meier curve represents the animal survival time from the beginning of tumour cell implantation. *P* value of <0.05 was considered statistically significant. D, Haematoxylin and eosin staining of the T3M4 (WT and SC) cells implanted mouse pancreas tumour tissues (left panel) and IHC analysis of STn antigen expression by VVA lectin staining (right panel)

**Figure 5:**
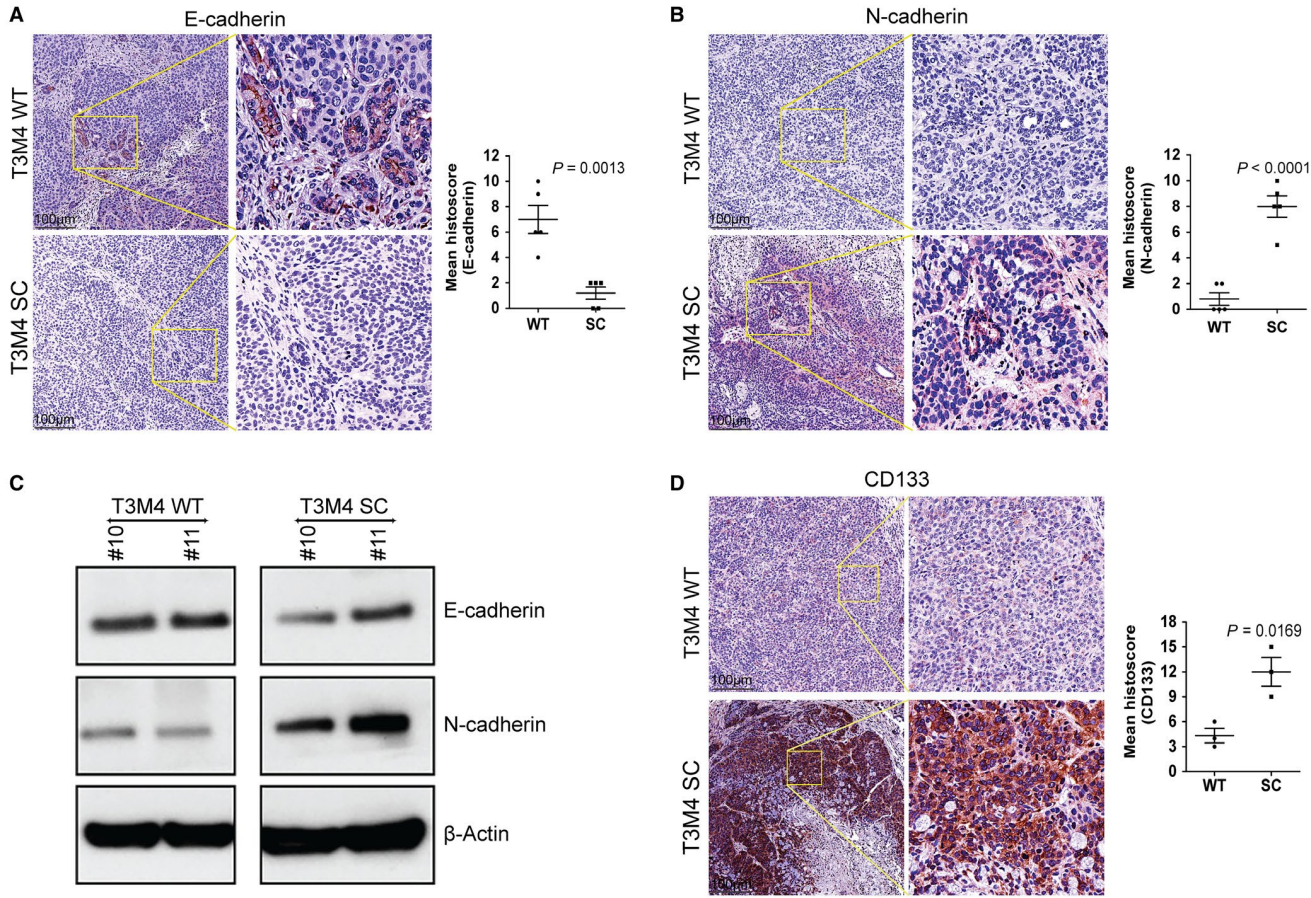
Analysis of EMT in orthotopic PDAC tumours. A, IHC analysis of E‐cadherin in T3M4 WT and SC cells implanted mouse pancreas tumour tissues. The mean histoscore of E‐cadherin expression was compared between WT and SC cells implanted mouse tumour tissues (n = 5). B, IHC analysis of N‐cadherin in T3M4 WT and SC cells implanted mouse pancreas tumour tissues. The mean histoscore of N‐cadherin expression was compared between WT and SC cells implanted mouse tumour tissues (n = 5). C, Western blotting of E‐cadherin and N‐cadherin expressions in T3M4 WT and SC cells implanted mouse pancreas tumour tissues. Detection of β‐actin served as loading control. *P* value of <0.05 was considered statistically significant. D, IHC analysis of the expression of CD133 in T3M4 WT and SC cell implanted mouse tumour tissues. The mean histoscore of CD133 expression was compared between WT and SC cells implanted mouse tumour tissues (n = 3). *P* value of <0.05 was considered statistically significant

We sought to assess the biological impact of truncated O‐glycans on tumour growth, metastasis and animal survival using a murine xenograft model, where T3M4 WT and SC cells were orthotopically implanted into the pancreas of athymic nude mice. Animals implanted with SC cells that expressing truncated O‐glycans showed a significantly increased tumour weight (*P* < 0.0001) (Figure 4A) and tumour volume (*P* < 0.0001) (Figure 4B) as compared to WT cells implanted tumours. Incidence of metastasis to liver (15.4%), lymph node (30.7%), spleen (30.7%), peritoneum (46.1%) and intestine (30.7%) was found to be high in T3M4 SC implanted tumours when compared with WT implanted tumour (Figure S2A). Remarkably, animals bearing T3M4 SC tumours showed a significantly reduced median survival of 51 days (*P* = 0.0002, log‐rank) as compared to animals bearing T3M4 WT tumours, which showed a median survival of 87 days (Figure 4C). Further, the immunohistochemical analysis of the lectin isolated from Vicia Villosa (VVA) in tumour tissues demonstrated that the abundant presence of Tn/STn antigens in T3M4 SC tumours as compared to WT tumours (Figure 4D). In order to delineate the increased incidence of metastasis upon implantation of truncated O‐glycan‐expressing T3M4 cells, we have analysed the expression of EMT markers such as E‐cadherin and N‐cadherin in mouse tumour tissue samples. Expression of E‐cadherin was prominent in WT cell implanted tumours, while negligible expression was observed in SC cells implanted tumours (*P* = 0.0013) (Figure 5A). In contrast to this, we detected an increased expression of N‐cadherin in SC cells implanted mice tumour tissues, whereas its expression was little in WT cells implanted mice tumour tissues (*P* < 0.0001) (Figure 5B). Also, we validated these results by Western blotting; expectedly, a more prominent cadherin switching with decreased expression of E‐cadherin and increased expression of N‐cadherin was observed in SC cells implanted tumours (Figure 5C). In addition to this, we have analysed the expression of CD133 in mice tumour tissues. More interestingly, the protein expression of CD133 was significantly high in SC cells implanted mice tumours as compared to WT cells implanted tumours (*P* = 0.0169) (Figure 5D). Taken together, these results suggest that aberrant expression of truncated O‐glycan enhances the tumour growth and metastasis, and reduces the tumour‐bearing animals survival through the induction of EMT and stemness features in PDAC cells.

## 4 DISCUSSION

Aberrant glycosylation and altered O‐glycan biosynthesis machinery have been implicated with the progression of several cancers including PDAC.^24^, ^25^ Incomplete O‐glycosylation results in the expression of truncated O‐glycans such as Tn antigen and its sialylated form STn antigen whose expression is highly restricted in normal tissues but is found only on tumour tissues.^26^, ^27^, ^28^, ^29^ The presence of truncated O‐glycans in cell surface of tumour tissues is highly associated with tumour progression, metastasis, chemoresistance and poor prognosis which makes STn antigen as an attractive therapeutic target.^30^, ^31^, ^32^ However, the key question that how COSMC deletion induced truncated O‐glycans enhance the tumorigenic potential of PDAC remains unanswered and was the major objective of the current investigation.

Though lot of evidence supporting the important role of glycosylation in inducing metastasis and acquiring drug resistance phenotype,^33^, ^34^, ^35^ none of the work has yet demonstrated the link that COSMC deletion mediated aberrant glycosylation induces tumorigenesis and metastasis through the induction of EMT and cellular plasticity in epithelial cells. In view of our previous study where we found the importance of COSMC and glycosylation machinery in the malignant phenotype of cancer cells and development of metastasis, and also other documented examples,^9^, ^36^, ^37^, ^38^ we hypothesized the possibility of aberrant glycosylation induced cellular phenotypic changes which enable the cells to efficiently invade the ECM and locate to distant organ sites. During EMT process, the epithelial cells which are closely connected to each other lose their apical to basal polarity, change the morphology to more elongated spindle shape, exhibits reduced expression of epithelial proteins and increased expression of mesenchymal proteins which result in the increased motility of the newly transformed cell.^39^, ^40^, ^41^, ^42^ Fundamental features of EMT can be specified by the expression of specific epithelial as well as mesenchymal marker proteins. E‐cadherin is typically found on epithelial cells, while mesenchymal cells express various cadherins, including N‐cadherin and R‐cadherin.^43^ In this study, an increased expression of N‐cadherin which is a non‐epithelial cadherin, in COSMC deleted epithelial PDAC cells (T3M4 SC and Capan‐2 SC) and subsequent loss of E‐cadherin, gives a strong indication for the induction of EMT. Negative expression of E‐cadherin and a strong positive expression of N‐cadherin, so‐called ‘cadherin switch’ have been reported in undifferentiated tumours and metastases.^44^, ^45^ Loss of claudin and occludin which are the integral membrane proteins localized at the epithelial tight junctions further support the induction of EMT upon COSMC deletion in epithelial PDAC cells. Along with N‐cadherin, other mesenchymal markers such as vimentin and the transcription factors Snail and Slug were also found to be up‐regulated in COSMC deleted truncated O‐glycan‐expressing PDAC cells. The transcription factor Snail directly suppresses E‐cadherin activity and such dysfunction of E‐cadherin results in the loss of epithelial cell polarity has already been reported,^46^, ^47^ which is in accordance with our findings. Convincingly, Cheng et al have identified the important role of MMPs, especially MMP2 in inducing EMT by degrading the basement membrane.^48^ In our study, we found an increased expression of MMPs and α‐SMA in COSMC deleted PDAC cells that endorsed the in vitro induction of EMT in PDAC cells that are expressing truncated O‐glycans. In agreement with our in vitro findings, a ‘cadherin switch’ where the decreased expression of E‐cadherin and increased expression of N‐cadherin was also observed in COSMC deleted cells implanted mouse tumour tissues. However, a detailed understanding of in vivo expression patterns of EMT proteins are necessary to conclude the close association between EMT and COSMC deletion mediated aberrant glycosylation induced tumorigenesis and metastasis, and that work is in progress.

**Figure 6:**
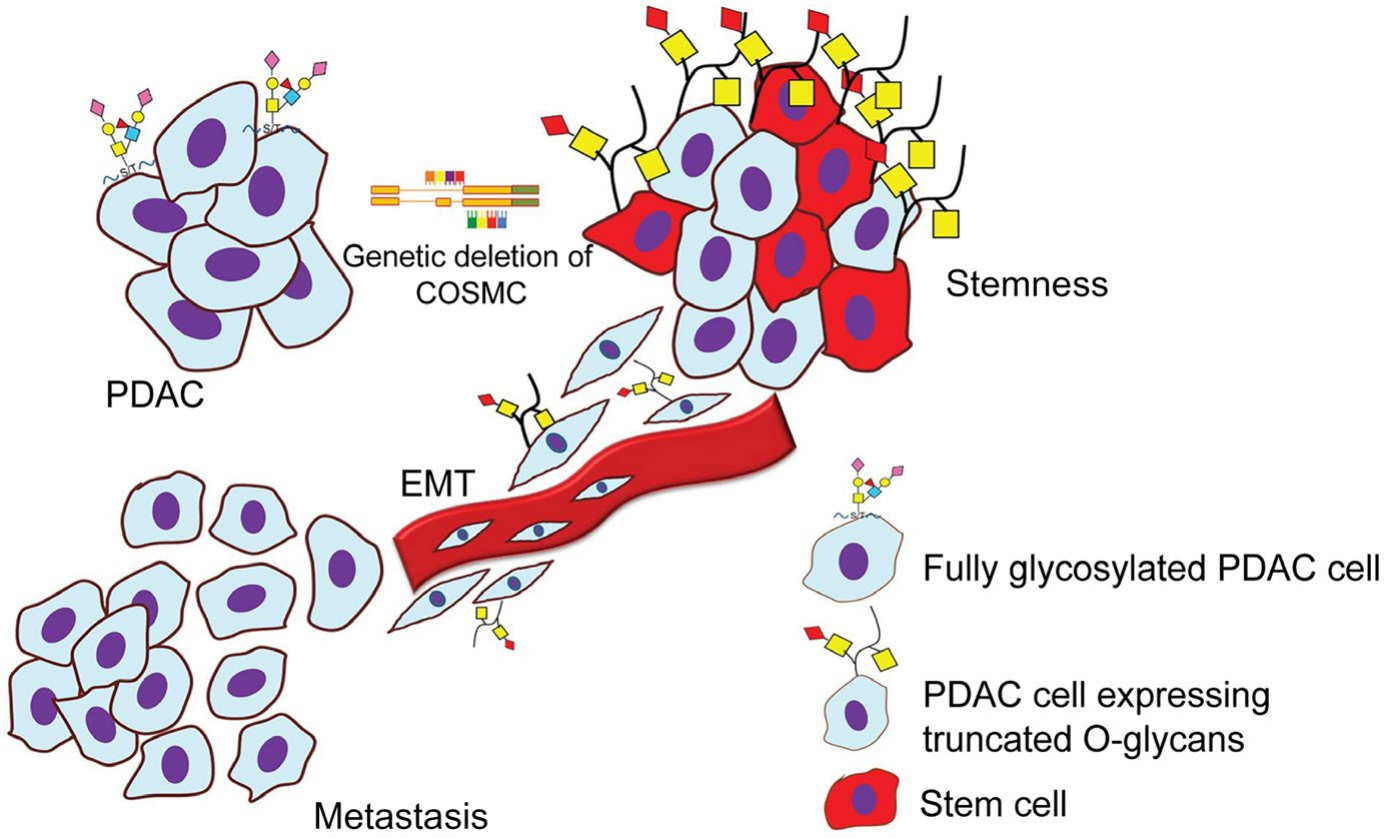
Proposed mechanism of truncated O‐glycans induced tumorigenicity in PDAC cells. Genetic deletion of COSMC results in the aberrant expression of Tn/STn antigen on mucin and other glycoproteins in PDAC cells. Such truncated O‐glycan‐expressing PDAC cells increase tumorigenicity and metastasis through the induction of EMT. Also, truncation of O‐glycans enhanced the cellular plasticity and stemness in PDAC cells

It is well understood that mucins play a protective role on epithelial surfaces and exert significant control on cellular signalling. However, mucins carrying aberrant O‐glycophenotypes in cancer cell surface profoundly exhibit tumour‐promoting effects, and our study gives a strong evidence for the first time, that COSMC deleted truncated O‐glycan carrying cancer cells promote tumorigenesis and metastasis through the induction of EMT. Yet, another intriguing finding of our current study is that, we found COSMC deletion enhances side population cells (SP) and cell surface stem cell markers CD44 and CD133 in PDAC which possesses stem cell‐like feature within the tissues. The mechanistic basis of overexpression of CD44/CD133 in truncated O‐glycan‐expressing PDAC cells is uncertain. However, Bidlingmaier et al^49^ have demonstrated that glycosylation status of CD133 plays a critical role in maintaining stem cell features. Yet, another study showed that sialylation regulates CD133 stability in cancer cells.^50^ Also, very recently, co‐expression of CD133 with STn antigen in a subset of human ovarian cancer cells has been reported.^51^ Interestingly, a recent study has demonstrated that CD44 is one of the major carriers of truncated O‐glycans which is accompanied by increased hyaluronan binding potential that affects matrix shedding.^52^ Altogether, these studies strongly support our hypothesis that aberrant glycosylation plays a critical role in maintaining the stemness features of cancer cells that may promote the induction of EMT (Figure 6). However, further studies are warranted to prudently elucidate the underlying mechanism of aberrant glycosylation in inducing stemness and EMT in cancer cells.

## CONFLICT OF INTEREST

The authors of this manuscript declare no conflicts of interest.

## AUTHOR CONTRIBUTION

Conception and design: DT and PR. Performed the experiments: DT, SS and TC. Analysed the data: PMG and PR. Wrote the paper: DT and PR. Final approval of the manuscript: DT, SS, TC, PMG and PR.

## Data Availability

The data that support the findings of this study are available from the corresponding author upon reasonable request.
