# Supplementary material for: Truncated O‐glycans promote epithelial‐to‐mesenchymal transition and stemness properties of pancreatic cancer cells
Source: J Cell Mol Med. 2019 Aug 7;23(10):6885–96. doi: 10.1111/jcmm.14572 (PMC6787448; doi:10.1111/jcmm.14572)

**Supplementary Information**

**Supplementary Table 1**

| Gene | Primer |
| --- | --- |
| COSMC | 5’-CACTGTGACAAAGCAGA-3’ (F)<br>5’-GGTTGGGGTGATAAGTCA-3’ (R) |
| C1GALT1C1 | 5’-GCGGCTGCCCAGAGAAGCAA-3’ (F)<br>5’-AGGGCCGGTCATAACCCAGCA-3’ (R) |
| GAPDH | 5’-GCCTTCCGTGTCCCCACTGC-3’ (F)<br>5’-GCTCTTGCTGGGGCTGGTGG-3’ (R) |

**List of cDNA specific PCR primers used for the study**

**Supplementary Table 2**

**Details of antibodies used for the study**

| Anti-body | Host species | Vendor | Cat.No | Dilution |
| --- | --- | --- | --- | --- |
| C1GalT1 | Mouse | Santa Cruz Biotechnology, CA, USA | sc-100745 | 1:1000 |
| E-cadherin | Rabbit | Cell Signaling Technology, MA, USA | 24E10 | 1:1000 |
| Claudin | Rabbit | Cell Signaling Technology, MA, USA | D5H1D | 1:1000 |
| Occludin | Mouse | Santa Cruz Biotechnology, CA, USA | sc-133256 | 1:500 |
| αSMA | Rabbit | Cell Signaling Technology, MA, USA | D4K9N | 1:1000 |
| N-cadherin | Rabbit | Cell Signaling Technology, MA, USA | D4R1H | 1:1000 |
| Snail | Rabbit | Cell Signaling Technology, MA, USA | C15D3 | 1:1000 |
| Slug | Rabbit | Cell Signaling Technology, MA, USA | C19G7 | 1:1000 |
| Vimentin | Rabbit | Cell Signaling Technology, MA, USA | D21H3 | 1:1000 |
| MMP2 | Rabbit | Cell Signaling Technology, MA, USA | D4M2N | 1:2000 |
| MMP9 | Rabbit | Cell Signaling Technology, MA, USA | D6O3H | 1:1000 |
| GAPDH | Rabbit | Cell Signaling Technology, MA, USA | 14C10 | 1:5000 |

| | | | | |
|---|---|---|---|---|
| β-actin | Mouse | Cell Signaling Technology, MA, USA | 8H10D10 | 1:5000 |

**Supplementary Figure legends**

**Supplementary Figure 1. Quantification of Western blotting results.** The band intensity of each protein was analyzed by NIH ImageJ software and the fold change was calculated by normalizing with respective loading control (β-actin and GAPDH). Each experiments was performed in triplicate. Statistical significance between groups was analyzed by Two-way ANOVA. *p* value less than 0.05 was considered as statistically significant.

**Supplementary Figure 2**. A. The incidence of tumor metastasis was compared between T3M4 WT and SC cells implanted tumor bearing animals by the Fisher's two-tailed exact test.

## Supplementary Figure 1

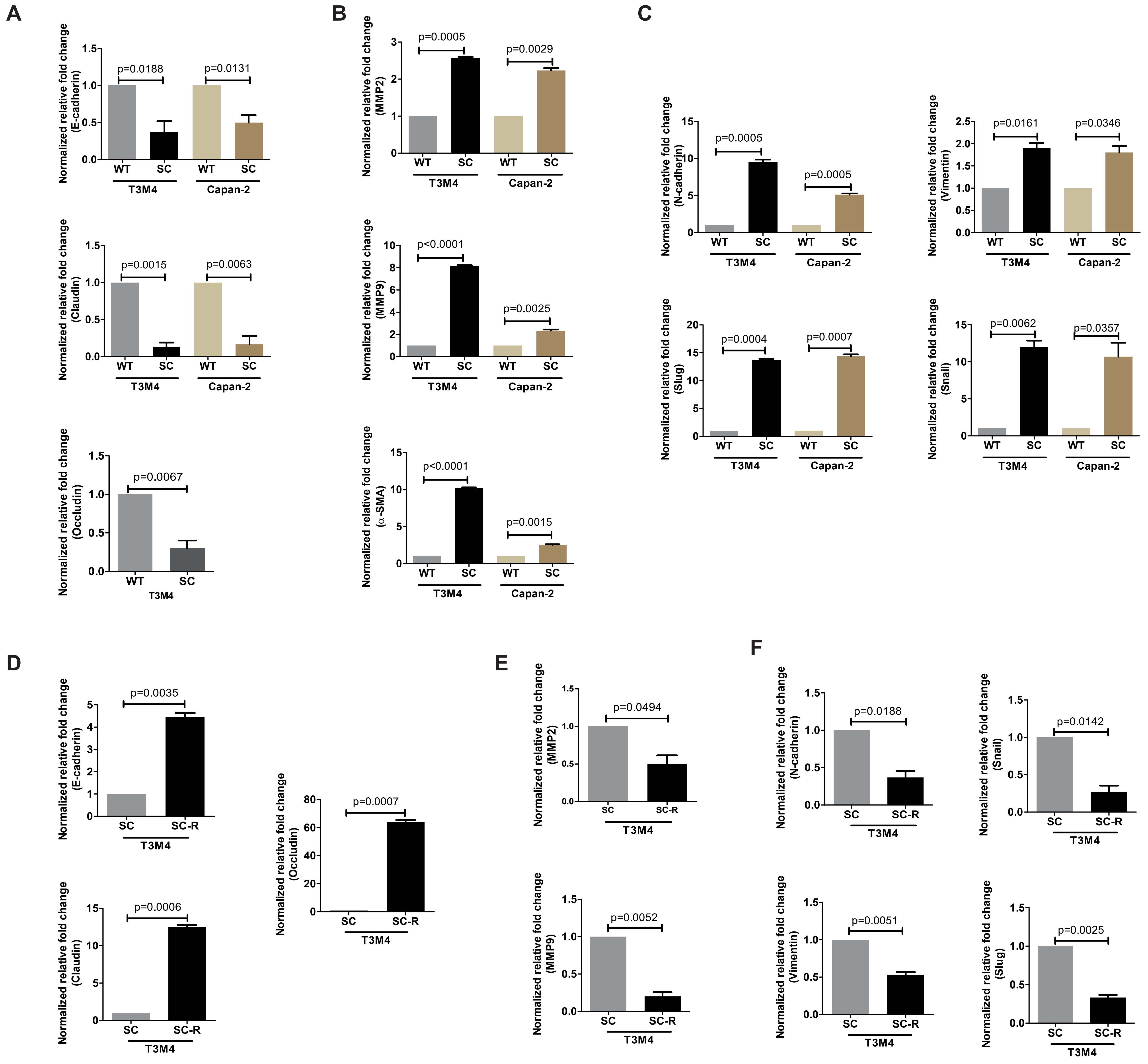

## Supplementary Figure 2

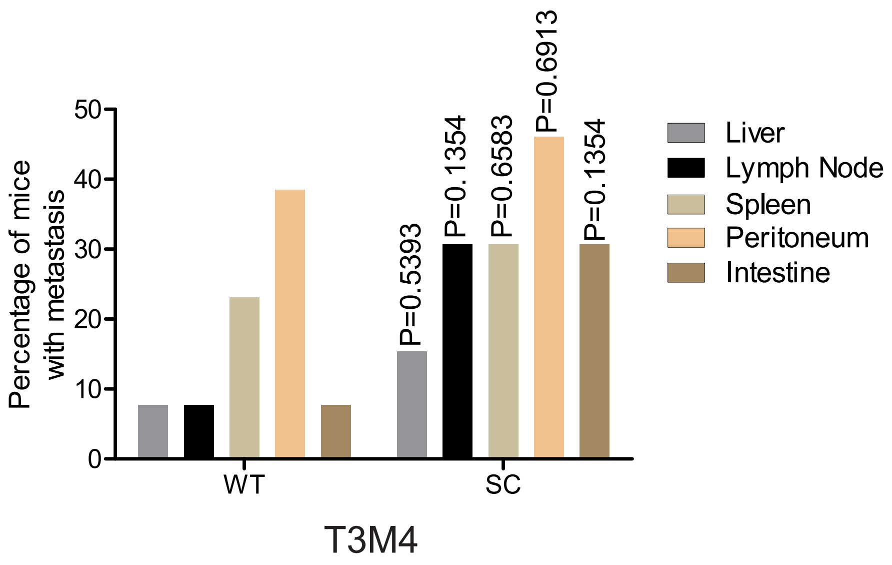

Supplement: Supplementary file 1 [file JCMM-23-6885-s001.pdf]
